# Extended law of laplace for measurement of the cloverleaf anatomy of the aortic root

**DOI:** 10.1007/s10554-023-02847-5

**Published:** 2023-04-12

**Authors:** Ehsan Ban, Paris-Dimitrios Kalogerakos, Ramak Khosravi, Bulat A. Ziganshin, Hesham Ellauzi, Abhay B. Ramachandra, Mohammad A. Zafar, Jay D. Humphrey, John A. Elefteriades

**Affiliations:** 1 Department of Biomedical Engineering, Yale University, New Haven, USA; 2 Aortic Institute, Yale School of Medicine, CB-3, 789 Howard Ave., New Haven, CT 06510 USA

**Keywords:** Aorta, Aortic root, Sinuses of Valsalva, Aortic diameter, Computerized tomography

## Abstract

The cross-sectional shape of the aortic root is cloverleaf, not circular, raising controversy regarding how best to measure its radiographic “diameter” for aortic event prediction. We mathematically extended the law of Laplace to estimate aortic wall stress within this cloverleaf region, simultaneously identifying a new metric of aortic root dimension that can be applied to clinical measurement of the aortic root and sinuses of Valsalva on clinical computerized tomographic scans. Enforcing equilibrium between blood pressure and wall stress, finite element computations were performed to evaluate the mathematical derivation. The resulting Laplace diameter was compared with existing methods of aortic root measurement across four patient groups: non-syndromic aneurysm, bicuspid aortic valve, Marfan syndrome, and non-dilated root patients (total 106 patients, 62 M, 44 F). (1) *Wall stress*: Mean wall stress at the depth of the sinuses followed this equation: Wall stress = BP × Circumscribing circle diameter/(2 × Aortic wall thickness). Therefore, the diameter of the circle enclosing the root cloverleaf, that is, twice the distance between the center, where the sinus-to-commissure lines coincide, and the depth of the sinuses, may replace diameter in the Laplace relation for a cloverleaf cross-section (or any shaped cross-section with two or more planes of symmetry). This mathematically derived result was verified by computational finite element analyses. (2) *Diameters*: CT scan measurements showed a significant difference between this new metric, the Laplace diameter, and the sinus-to-commissure, mid-sinus-to-mid-sinus, and coronal measurements in all four groups (*p-value* < 0.05). The average Laplace diameter measurements differed significantly from the other measurements in all patient groups. Among the various possible measurements within the aortic root, the diameter of the circumscribing circle, enclosing the cloverleaf, represents the diameter most closely related to wall stress. This diameter is larger than the other measurements, indicating an underestimation of wall stress by prior measurements, and otherwise provides an unbiased, convenient, consistent, physics-based measurement for clinical use.

**Graphical abstract:**

“Diameter” applies to circles. Our mathematical derivation of an extension of the law of Laplace, from *circular* to *cloverleaf* cross-sectional geometries of the aortic root, has implications for measurement of aortic root “diameter.” The suggested method is as follows: (1) the “center” of the aortic root is identified by drawing three sinus-to-commissure lines. The intersection of these three lines identifies the “center” of the cloverleaf. (2) The largest radius from this center point to any of the sinuses is identified as the “radius” of the aortic root. (3) This radius is doubled to give the “diameter” of the aortic root. We find that this diameter best corresponds to maximal wall stress in the aortic root. Please note that this diameter defines the smallest circle that completely encloses the cloverleaf shape, touching the depths of all three sinuses.

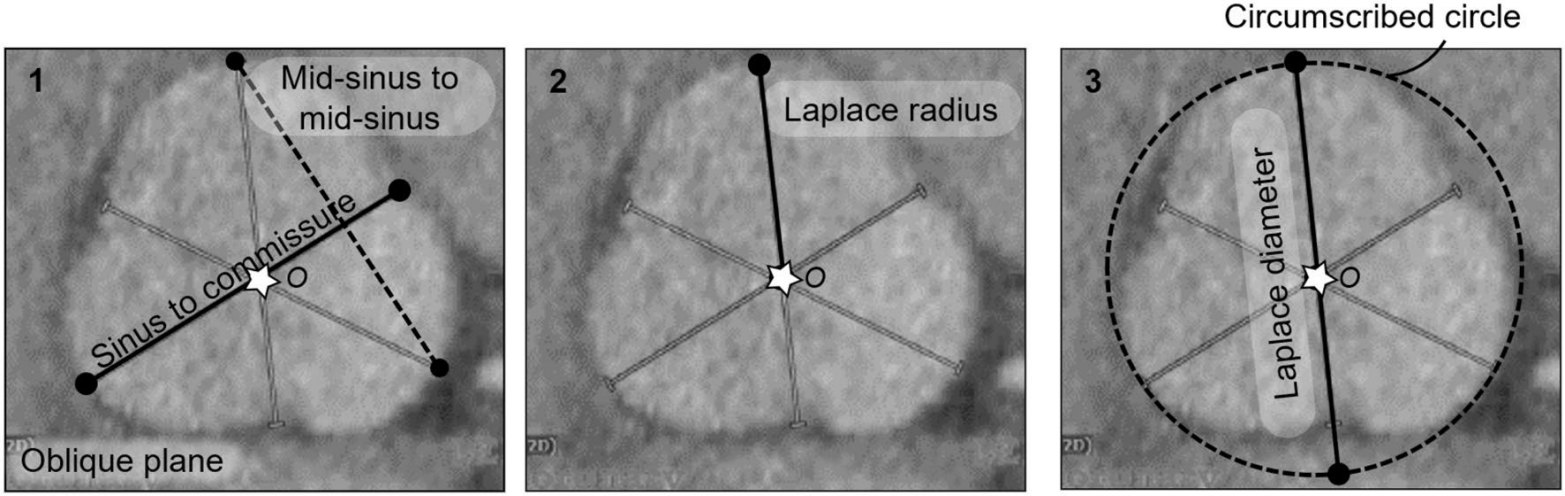

**Supplementary Information:**

The online version contains supplementary material available at 10.1007/s10554-023-02847-5.

## Introduction

The ultimate fate of a compromised thoracic aorta—dilatation, dissection, and rupture—depends on the imposed hemodynamic loads and mechanical properties of the wall, that is, on the mechanics. Of particular importance is a measure of the intensity of the force within the aortic wall, often calculated as a mechanical stress. Assessing and understanding values of wall stress are important clinically and scientifically for two key reasons: the process of aneurysmal dilatation depends in part on mechanobiological responses of vascular cells to changes in wall stress, and dissection and rupture occur when wall stress exceeds wall strength [1, 2]. Advances in medical imaging and computational methods now enable detailed calculations of wall stress in the aorta [3–5], but these calculations require knowledge of patient-specific mechanical properties, which evolve as the disease progresses and vary from region-to-region even under normal conditions. Despite continued advances, it remains difficult to estimate the requisite (nonlinear, anisotropic) mechanical properties of the aorta.

For this reason, many rely on the classical law of Laplace, found prominently in engineering and medical textbooks alike. Although the simplicity of this relation, that wall stress is determined by blood pressure, inner diameter, and wall thickness, renders it attractive for use, this relation strictly holds only for an idealized cylindrical geometry. Another seldom appreciated advantage of this relation is its universality: it holds independently of the underlying mechanical properties of the material since it is derived from a force balance (equilibrium) alone. For this reason the law of Laplace always provides the correct mean (radially averaged) value of wall stress in the circumferential direction of a pressurized cylindrical tube [6]. Yet, the complex, non-cylindrical cross-sectional geometry of the aortic root necessitated a search for an *extended* law of Laplace.

Currently, measurements of aortic diameter critically influence decisions on surgical aortic intervention. There is, however, a “dirty little secret” in radiographic imaging and measurement of the aorta: it is unclear how best to measure this most anatomically complex site within the aorta, the aortic root [7]. Measuring the simple diameter of the aorta at different positions—ascending, arch, descending, and abdominal—is reasonable only because the lumen is circular in cross-section. By contrast, the aortic root has a 3-lobed cloverleaf cross-sectional shape. There is no definition for the diameter of such a structure, hence rendering the classical law of Laplace inappropriate for use at the aortic root.

**Fig. 1:**
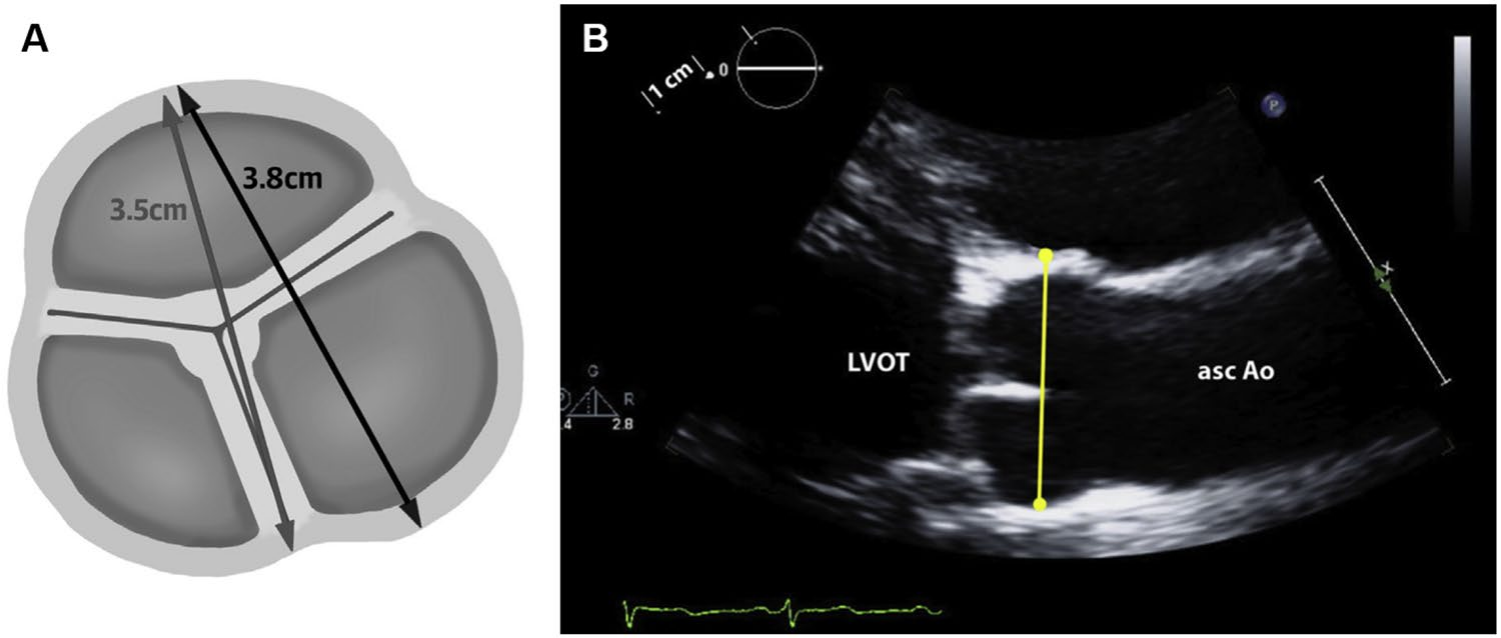
**A** Comparison of (lighter-colored line) “sinus-to-commissure” and (darker-colored line) “sinus-to-sinus” methods of measurement of “diameter” of the aortic root. Reproduced (modified) with permission from Elefteriades JA, Mukherjee SK, Mojibian H. Discrepancies in measurement of the thoracic aorta. JACC. 76(2);2020:201–17. **B** Note that the imaging plane in this ECHO has visualized both sinuses “full,” not off-axis, misrepresented by a “flat” appearing sinus contour. Reproduced with permission from Goldstein SA, Evangelista A, Abbara S, et al. Multimodality imaging of diseases of the thoracic aorta in adults: from the American Society of Echocardiography and the European Association of Cardiovascular Imaging. J Am Soc Echocardiogr. 2015;28(2):119–82

Absent societal guidelines, different imaging centers measure the aortic root differently (Fig. 1A). Many centers measure a “diameter” from the depth of a sinus to the opposing commissure; other centers measure a “diameter” from the depth of one sinus to the depth of the opposing sinus. Furthermore, for each of these methods, there are three potential measurements, one for each sinus. If all three sinuses are indeed measured, most centers would likely choose the largest of the three measurements for the representative diameter. Clearly, then, there can be substantial differences between measurements, with the sinus-to-sinus technique yielding larger measured dimensions. Without consistent techniques, accuracy of follow-up and precision of clinical care will suffer. Furthermore, without consistent techniques, research studies—especially those linking aortic size to adverse clinical outcomes—will also suffer.

One may wonder how echocardiography (ECHO) of the aortic root fits into this paradigm. As rightly taught by experts, the echocardiographer must ensure that both sinuses are full in the images selected for measurement. The plane of the image must be well selected to achieve this goal (Fig. 1B). Anatomically, this corresponds most closely to a sinus-to-sinus image on CT or MRI. Not achieving an ECHO image with full sinuses will underestimate the true sinus-to-sinus dimension [8].

With these many considerations in mind, we sought to explore from a bioengineering standpoint the question of what method of measurement of aortic root diameter is more closely related to wall stress and thus preferred for clinical assessment. This paper presents the product of these efforts. It includes (i) a mathematical extension and validation of the law of Laplace from a circular to a cloverleaf cross-sectional shape, and (ii) careful assessment of patient imaging studies to determine which method of aortic root measurement best represents wall stress by the extended Laplace formula. In this way, we identified a scientifically motivated definition for the diameter of the clover-leaf shaped cross-section of the aortic root.

## Methods

This study was approved by the Human Investigations Committee of Yale University.

### An extended Laplace relation for the clover leaf-shaped sinuses of Valsalva

We used a standard balance of forces (equilibrium) and the symmetrical properties of a prototypical aortic root to derive mathematically an extended law of Laplace for a cloverleaf shaped cross-section at the sinuses of Valsalva. Interpretation of this mathematical derivation is limited to the two-dimensional oblique plane of the root.

**Fig. 2:**
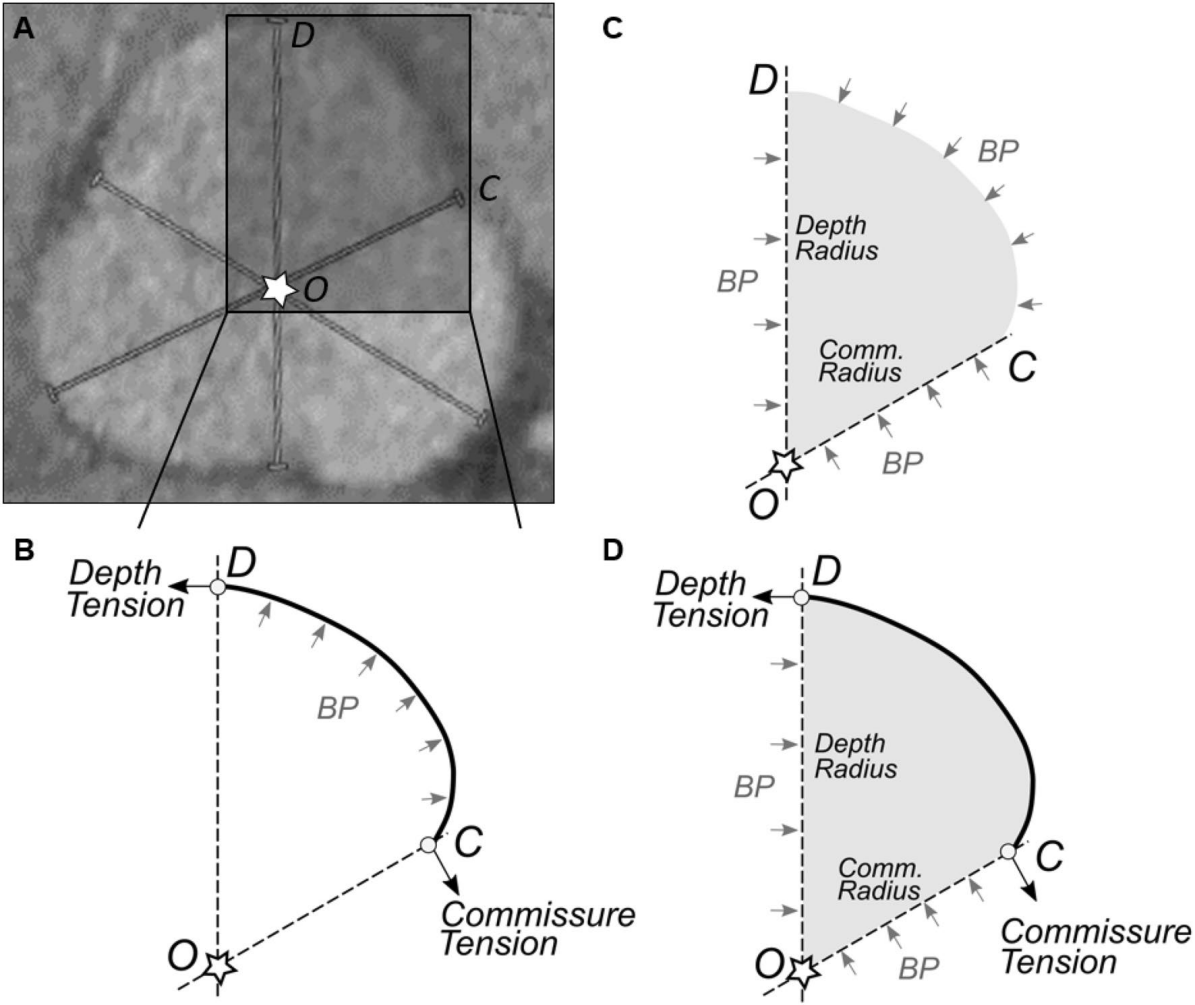
Derivation of an extended Laplace relation for shapes having multiple planes of reflective symmetry. **A** The depth of a sinus, $D$, commissure $C$, and the center, $O$, where the planes of symmetry coincide, are used in the derivation of the extended law of Laplace, Eq. (1), as described in Supplemental Materials. **B** The free body diagram, including a portion of the wall and blood, used in deriving the extended law of Laplace

If we define the center of cloverleaf shaped cross-section as the point where the planes of symmetry, or the three depth-to-commissure lines, coincide (at Point *O* in Fig. 2), then the radially-averaged wall stress can be calculated as (see Supplementary Information for a description of the mathematical derivation)

1 $$ {\text{Wall}}\;{\text{stress}} \cong {\text{Pressure}} \times \frac{{{\text{Distance}}\;{\text{from}}\;{\text{wall}}\;{\text{to}}\;{\text{center}}}}{{{\text{Wall}}\;{\text{thickness}}}}, $$

which holds for any cross-sectional shape with two or more planes of reflective symmetry. Equation (1) thus reveals that maximum wall tension occurs at the depth of a sinus independent of the specific material properties. By considering the similarity of Eq. (1) to Laplace’s law for a circular section, the equivalent diameter in a cloverleaf shaped cross-section is defined as twice the distance from sinus depth to cloverleaf center, which equals the diameter of a circle that circumscribes the cloverleaf. Note that these dimensions emerged naturally from the mathematical derivation; they were not selected based on prior experience or hypothesis.

The validity of this extended law of Laplace, derived from first principles, was assessed by comparison to results from finite element analyses using two different computational programs (commercially available and open source), two different material models (linear and nonlinear), and different cross-sectional geometries and distending pressures (see Supplementary Information). It can also be seen that this relationship reduces to the classical law of Laplace for circular cross-sections, with 2 × distance to the center equal to the circle’s diameter, which provides additional validation.

### Patients

We next considered clinical data with patients (n = 106) divided into four groups: those with non-dilated aortic roots (controls), non-syndromic root dilation, Marfan syndrome (MFS), and bicuspid aortic valve (BAV). We sampled male and female patients by a 1 to 1 ratio, where possible. We used a root index [11], defined as mean depth to commissure measurement/patient height^2.7^, to delineate patients in the non-dilated group from those in the non-syndromic dilated group, where patient height was in meters. A cut-off value of 8.5 mm/m^2.7^ was used to categorize roots as non-dilated versus dilated. Patient characteristics are listed in Table 1.

**Table 1:** Patient characteristics

Patient characteristic	Patient groups/parameter values			
	Nondilated	Nonsyndromic dilated	Marfan syndrome	Bicuspid Aortic Valve
Number of patients	25	25	28	28
Age (years)	73 ± 2	74 ± 2	41 ± 3	60 ± 2
Male/Female	12/13	16/9	14/14	20/8
Height (cm)	177 ± 2	171 ± 2	185 ± 2	177 ± 2
Weight (kg)	86 ± 4	95 ± 6	85 ± 5	91 ± 4
Body surface area (m^2^)	2.03 ± 0.05	2.05 ± 0.05	2.07 ± 0.06	2.08 ± 0.05
History of hypertension	15	18	12	14
History of diabetes	4	2	1	5

Values are listed, as appropriate, as the mean ± standard error

### Statistical tests

Statistical tests were performed using GraphPad Prism (version 9, GraphPad Software Inc, San Diego, California). The Anderson–Darling test was performed to assess data normality. T-test and Mann–Whitney test were performed to compare two groups of measurements. One-way ANOVA test with Geisser-Greenhouse correction followed by Dunnett multiple comparisons test as well as Friedman tests with Dunn’s multiple comparisons test were performed to compare multiple groups. Differences with *p*-*values* < 0.05 were considered significant.

### Area measurements

The area of the cloverleaves was measured in the oblique plane using ImageJ (National Institutes of Health, Bethesda, Maryland; Ref. 12). An arbitrary measurement was marked on Visage (Visage Imaging, San Diego, California) that was then used for setting the image scale in ImageJ. The periphery of the cloverleaf shaped cross-section was subsequently marked by a polygonal selection, whose area was computed by ImageJ.

## Results

### Validation of the mathematical derivation by finite element computations

**Fig. 3:**
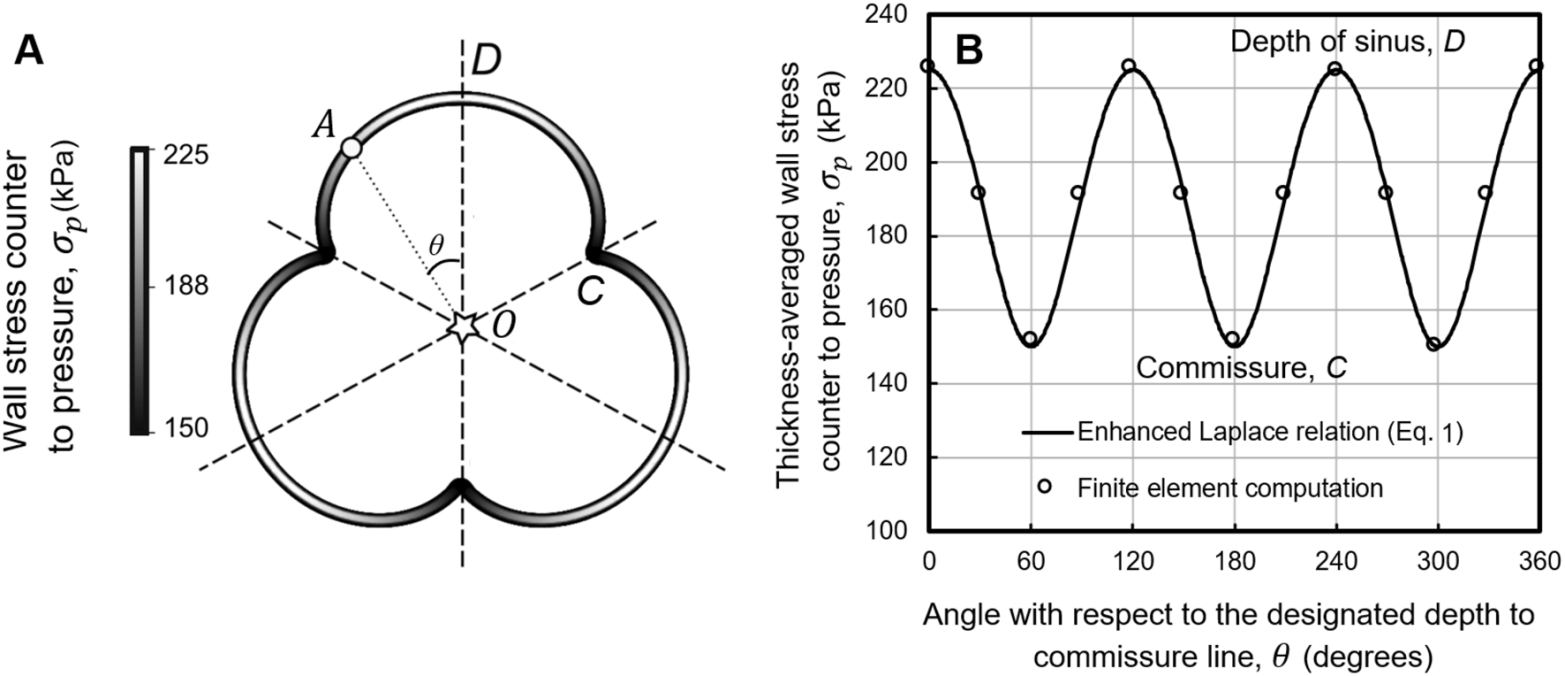
Validation of the extended Laplace relation for wall stress at the sinuses of Valsalva using finite element computations. The finite element numerical computations of maximum principal stress (open circles), calculated at multiple points around the perimeter of the cross-section, agree with the mathematically derived (solid curves) extended Laplace relation, Eq. (1). Computations using linear elastic and nonlinear material models produced the same result with high precision, confirming the universality of the extended law of Laplace

The extended Laplace relation was derived mathematically using a force balance and a fluid–solid free body diagram. This derivation was validated by comparing results with those obtained using finite element computational methods. The wall tension values, perpendicular to radii tracing outward from the center, computed by the finite element model equaled those from the extended Laplace relation (Eq. 1) with high precision in all tested cases (to within 0.01 of a percent), including that for an idealized epitrochoid shape of the aortic root (Fig. 3B). The agreement between the two methods, around the perimeter of the cross-section, was independent of the applied pressures over the range 80 to 150 mmHg. Moreover, the independence of the calculated wall stress from a material model and associated parameters in the finite element computations confirmed the universality of the extended Laplace relation (Eq. 1).

### Comparison of different measurements in the four patient groups

**Fig. 4:**
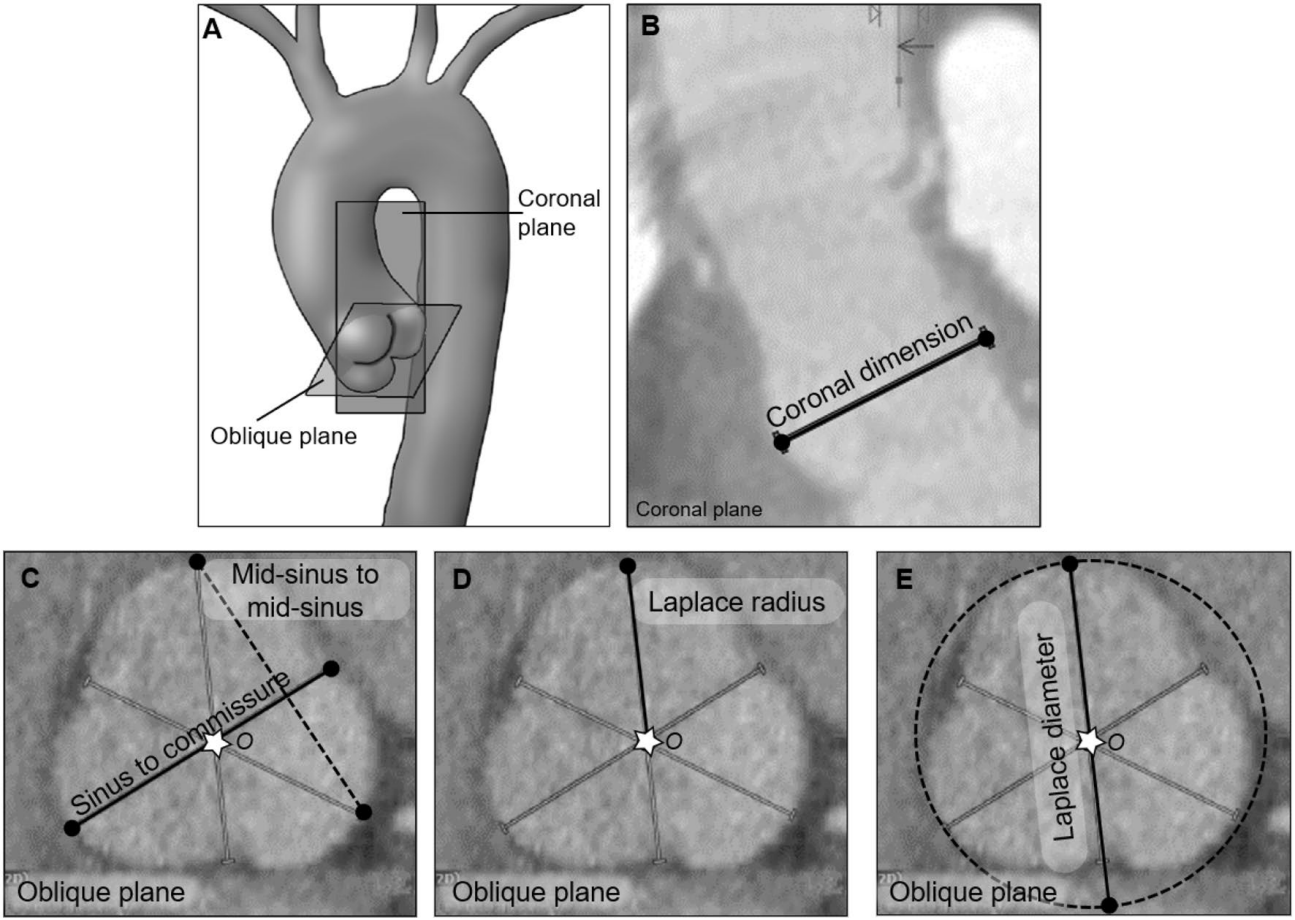
Various measurements of aortic root size at the sinuses of Valsalva. **A** Schematic drawing of the aorta with the coronal and double-oblique planes marked at the sinuses of Valsalva. **B** Coronal measurement of aortic size within the coronal plane. **C** Mid-sinus-to-mid-sinus (dashed line) and sinus-to-commissure (solid line) measurements within the oblique plane marked in panel (**A**). **D** Laplace radius is the distance from the center (star) of the cloverleaf, where the three sinus-to-commissure lines coincide, to the depth of a sinus. **E** The Laplace diameter, twice the Laplace radius, is the diameter of the smallest circle enclosing the cloverleaf shape, the circumscribed circle, which touches the depths of the three sinuses

**Fig. 5:**
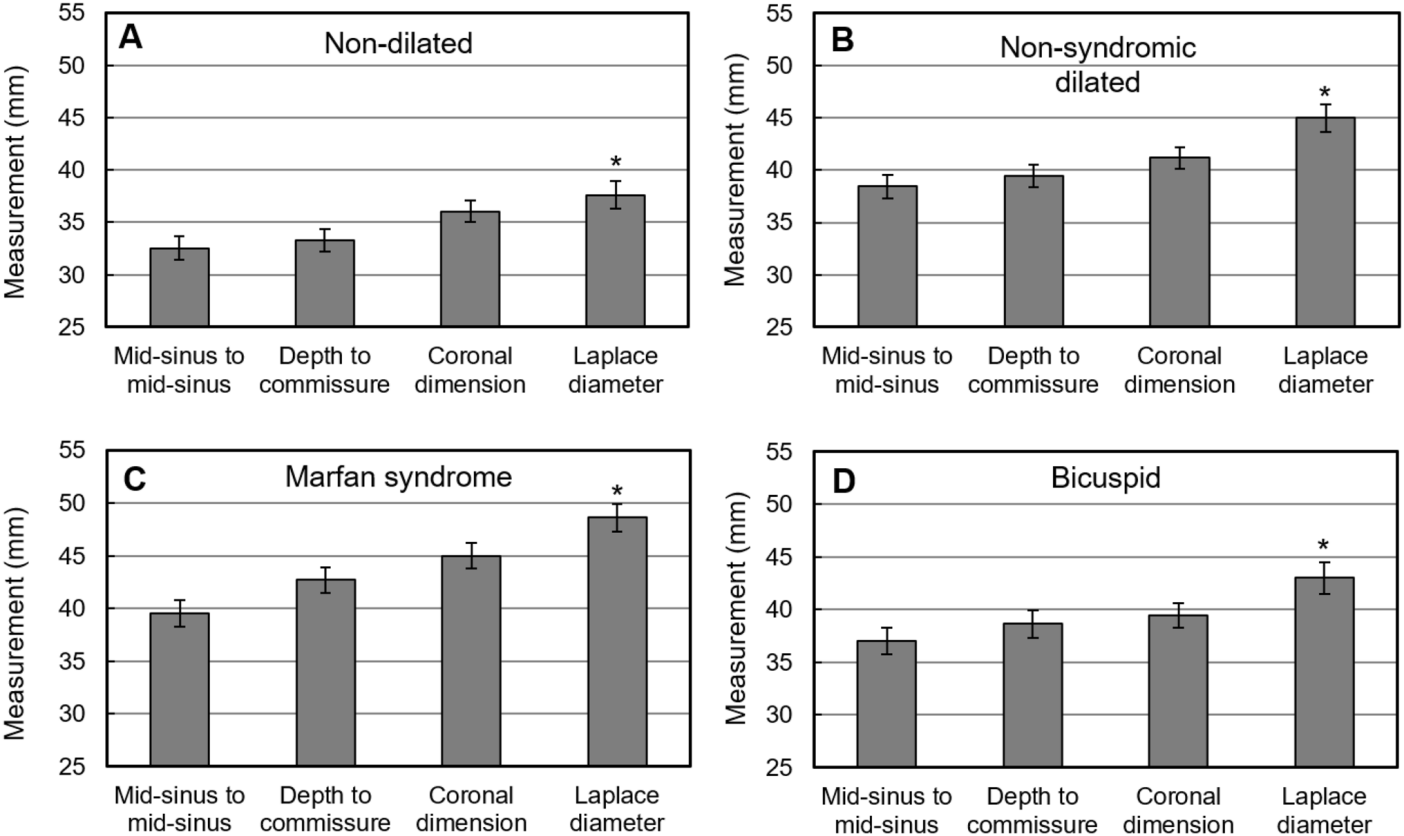
Comparison of the Laplace diameter with other measurements across four different patient groups suggests that wall tension is generally underestimated in the cloverleaf shape of the aortic root. The coronal dimension was measured as in Fig. 4. The error bars indicate standard errors. The Laplace diameter differed significantly (*) from each of the three other measurements in each of the four groups (*p-value* < 0.05)

We quantified the dimensions of aortic roots at the sinuses of Valsalva using previously employed metrics and the new proposed metric, the Laplace diameter (Fig. 4E). Measurements and associated comparisons across all patients are shown for each of the four groups (Fig. 5). Although the aortic roots were different in size across the four groups, the Laplace diameter was significantly different than each of the other metrics within each group (*p-value* < 0.05). The Marfan group exhibited the largest measured Laplace diameters within this cohort, followed by the non-syndromic dilated and then bicuspid aortic valve groups.

### Correlation of Laplace diameter with the area of the cloverleaf and patient height

**Fig. 6:**
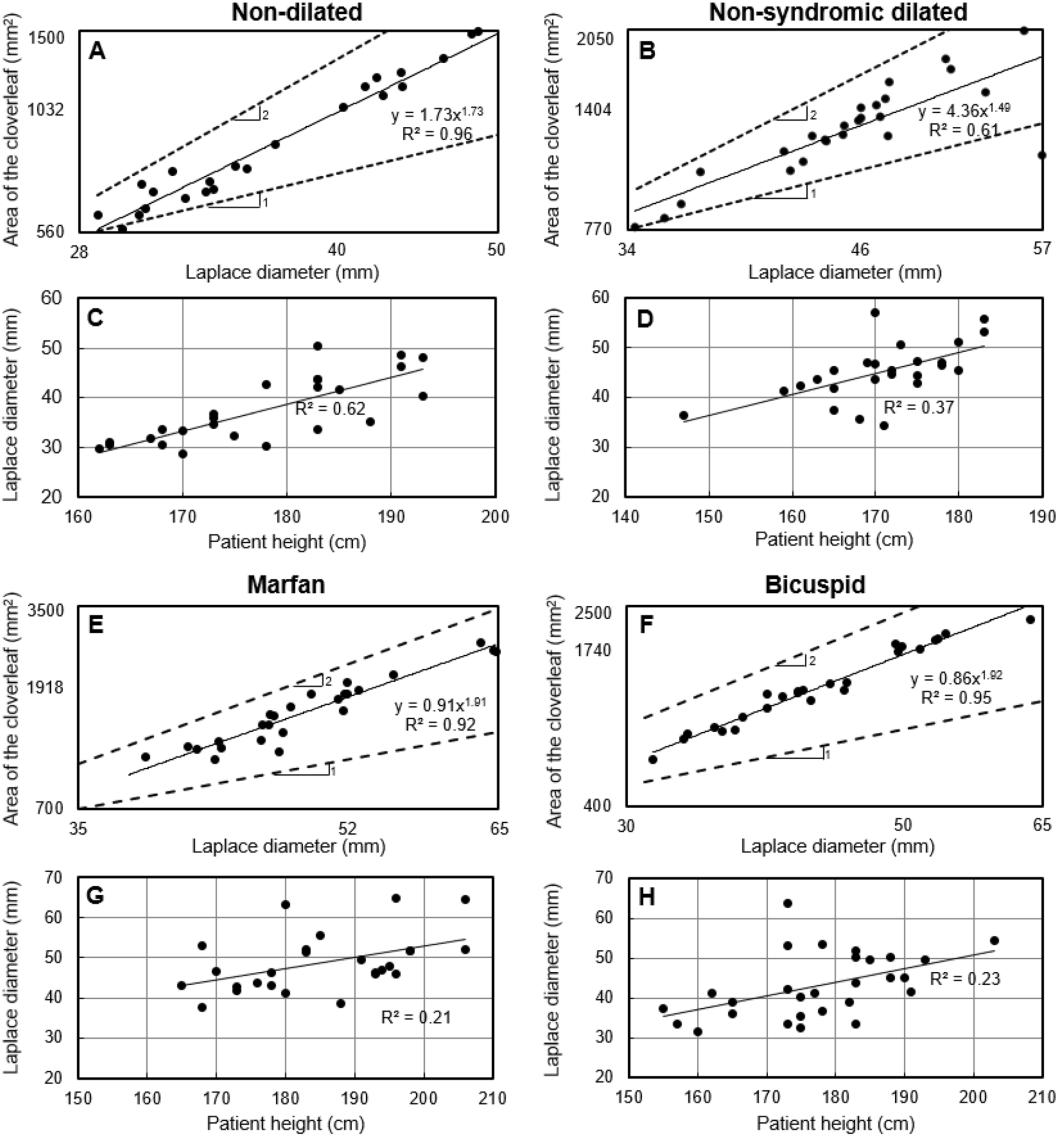
Variation of the Laplace diameter with either area of the cloverleaf or patient height in each of the four patient groups. The correlation between Laplace diameter and patient height is stronger in the non-dilated group as compared with that in the other three groups. In panels **A**, **B**, **E**, and **F**, both axes are plotted logarithmically. A few patients from the four groups were not included in the correlation analysis because the related data was unavailable at the time of this analysis. Note that the dashed lines are plotted to show power-law relations with exponents 1 (linear) and 2 (quadratic). The solid lines represent (**A**, **B**, **E**, and **F**) power-law and (**C**, **D**, **G**, and **H**) linear fits to the data

We next assessed possible correlations between the Laplace diameter and two readily measured patient-specific metrics: the area of the cloverleaf shaped cross-section at the root and patient height. Cloverleaf area was found to scale with Laplace diameter via a power-law with an exponent between 1 and 2 (Fig. 6). An exponent equal to 1 would indicate a linear correlation, whereas 2 would correspond to a uniform expansion of the cloverleaf with no change in shape. Observation of an exponent smaller than 2 agrees with the clinically observed change in shape of the cloverleaf shaped cross-section in more dilated roots. The linear correlations between the Laplace diameter and patient height were stronger in the non-dilated group (*R*^2^ = 0.62) than in the non-syndromic dilated, Marfan, and bicuspid groups (*R*^2^ = 0.37, 0.21, and 0.23, respectively).

### Effect of asymmetry of the sinuses in the bicuspid patients

**Fig. 7:**
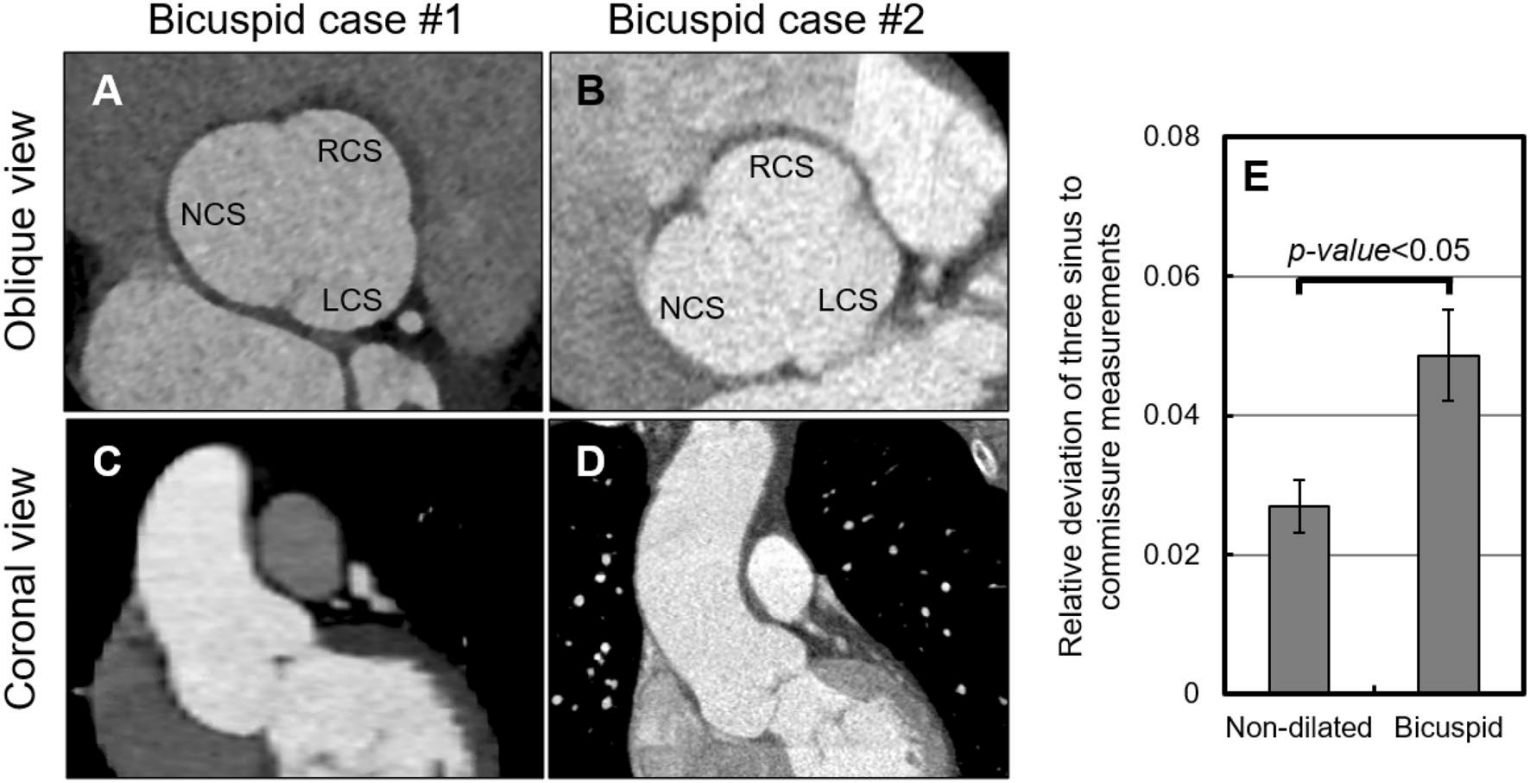
The difference of the measurements in the different cusps increases in asymmetrically dilated roots. The cloverleaf shape of the root appears more asymmetric in the group of patients with bicuspid aortic valves (two bicuspid cases shown in panels **A**–**D**) compared with the baseline, the non-dilated group. The non-coronary (NCS), left-coronary (LCS), and right-coronary sinuses (RCS) are marked in panels A and B

The derivation of the extended Laplace relation relies on the existence of two or more planes of symmetry within the cloverleaf shaped cross-section of the aortic root. These shapes were not all symmetric, however. For example, the symmetry condition was absent in roots where the cusps were not of the same size. Especially in the case of the bicuspid aortic valves, we visually observed a relatively larger number of asymmetrically dilated roots (Fig. 7). We thus quantified the asymmetric dilatation of the roots via a two-step approach. First, we found the mean value of the three sinus-to-commissure dimensions in a single root; second, we found the relative deviation of each measurement from the mean. The calculated relative deviations were significantly higher in the bicuspid group compared with baseline, the non-dilated group (*p-value* < 0.05), as seen in Fig. 7E. We point out that the assessed roots were only mildly asymmetric, and in these cases we expect the extended law of Laplace to reasonably estimate wall stress. Moreover, in case of an asymmetrically dilated root, a conservative estimate may be made using twice the distance between the center of the cloverleaf and the depth of the most dilated sinus as the diameter.

### Effect of the less protruded shape of the root in the Marfan syndrome patients

**Fig. 8:**
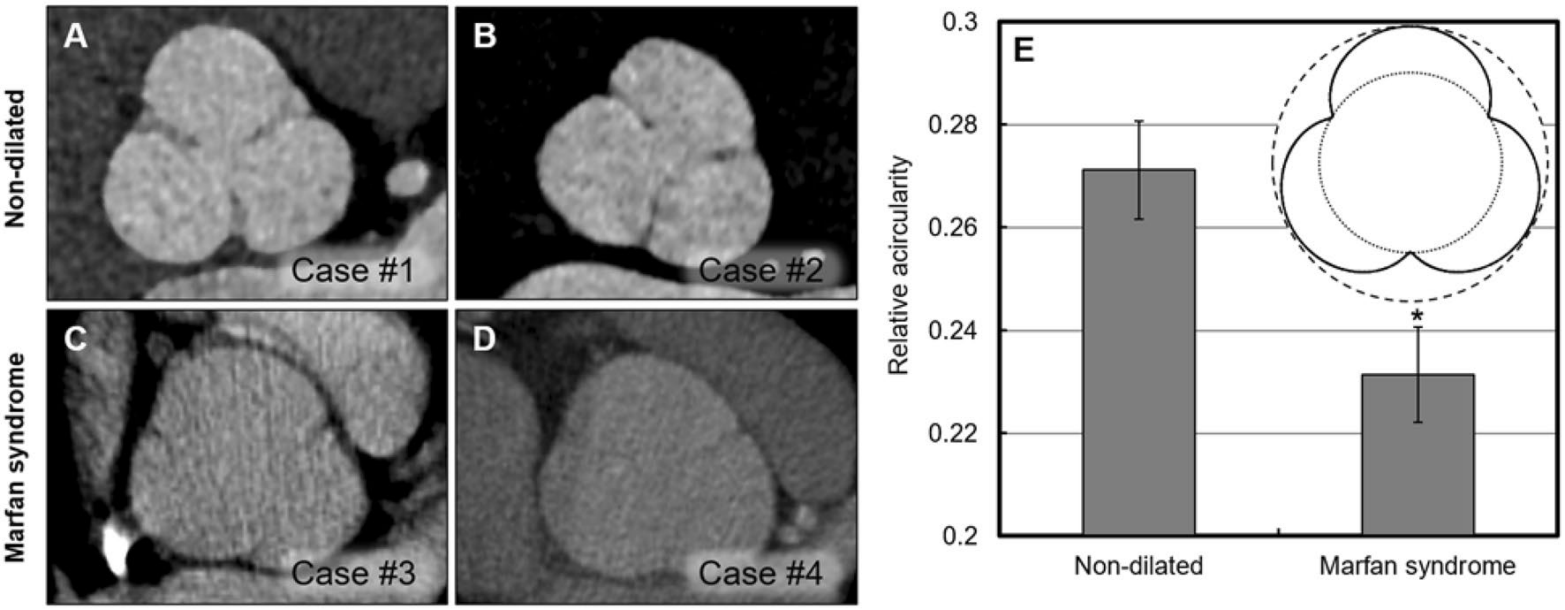
Differences between the Laplace diameter and the sinus-to-commissure measurements are smaller if the cloverleaf shape of the root appears more round and less protruded. A rounder shape is observed in the roots in Marfan syndrome. Snapshots of high acircularity versus circularity are shown in the cases of (**A** and **B**) non-dilated and (**C** and **D**) Marfan syndrome groups. **E** Quantitative comparison of acircularity between the non-dilated and Marfan groups, which differed significantly (*, *p-value* < 0.05). The *inset* displays the (dashed) circumscribed and (dotted) inscribed circles for an idealized root geometry. The difference in the diameters of the two circles was used in evaluating cloverleaf acircularity

The derivation of the extended law of Laplace suggested that the most dramatic differences between the Laplace diameter and sinus-to-commissure measurements should emerge in aortic roots with more protruded sinuses. Various degrees of protrusion presented in the set of assessed roots. For instance, in the Marfan group, roots frequently exhibited more round / circle-like shapes, with less protruded sinuses, compared with the non-dilated group, which included more protruded sinuses (Fig. 8). We quantified the differences in the protrusion of sinuses in these two groups by calculating acircularity of the cloverleaf shaped cross-sections at the root. Acircularity was measured by the relative difference in the diameters of the circles enclosed by and enclosing the cloverleaf shape (Fig. 8E, *inset*). A significant difference was observed in the relative acircularity of the non-dilated versus the Marfan roots (*p-value* < 0.05). That is to say, with the more severe deformity of the root in Marfan patients, the normal distinct sinus configuration (with normal, substantial protrusion of the sinuses) gave way to a more circular root shape.

## Real world clinical application

In order to assess clinical applicability and intra- and inter-observer reliability, we applied the Laplace measurement technique to the most recent 24 consecutive, unselected patients seen in consultation (office of JAE) who had high quality CT scans available for analysis (official radiology reports with aortic root measurements were available for 18 of the 24 patients).

### Comparative diameters by various techniques

In this group of patients, aortic root diameter by the Laplace technique ranged from 30.4 mm to 59.5 mm, with a mean of 41.6 mm. These Laplace diameters were larger than the root diameters measured in the standard radiology reports (range 27 mm to 47 mm, mean 37.3 mm). The conventional coronal aortic root measurements were larger than root diameters provided in radiology reports in 17 of the 18 patents (mean difference of 2.5 mm, range 0 to 4.1 mm). The Laplace diameters (above) were larger even than the coronal root diameters measured conventionally by our team (range 26.3 mm to 50.0 mm, mean 38.3 mm). Diameter of the aortic root in coronal views was measured by scrolling anteriorly and posteriorly and selecting the image with the largest diameter. Hence: Standard measurements < Coronal measurements < Laplace measurements.

### Intra-observer variability

Two observers (JAE and BAZ) independently measured the aortic roots by the Laplace technique on two separate occasions, one week apart. The absolute aortic root diameter differences between the two measurement sessions ranged from 0.2 mm to 2.4 mm, mean 0.9 mm for one observer (JAE) and between 0 and 2.2 mm, mean 0.6 for the other observer. The intra-observer variability, analyzed by the Intra-class Correlation Coefficient (ICC) [13] was 0.96 (JAE) and 0.97 (BAZ).

### Inter-observer variability

A second experienced reader (BAZ) read the Laplace diameters independently (of JAE). The absolute aortic root radius differences between the average of two measurements obtained by the two observers ranged from 0.2 to 2.9 mm (mean 1.6) (Table 2). The inter-observer variability ICC index was 0.89. Values of the Intra-class Correlation Coefficient (used in this analysis for inter- and intra-observer variability calculations) between 0.75 and 0.9 show good reliability, and any value above 0.9 indicates excellent reliability [13].

**Table 2:** Quantification of inter-observer variability in measurements

Measurement types	Mean		Range	
Radiology report measurements (mm) (n = 18)	37.3		27.0–47.0	
	Reader 1 (JAE)	Reader 2 (BAZ)	Reader 1 (JAE)	Reader 2 (BAZ)
Conventional Coronal Aortic Root Measurements (mm)	38.3	39.0	26.3–50.0	29.3–49.6
Laplace Aortic Root Measurements (diameter, mm)	41.6	44.4	30.4–59.5	33.0–57.6
Difference in Conventional Coronal Aortic Root Measurements between Reader 1 and Reader 2 (mm)	1.5		0–6.7	
Difference in Laplace Aortic Root Measurements (radius) between Reader 1 and Reader 2 (mm)	1.6		0.2–2.9	

## Discussion

Thoracic aortic aneurysm and dissection (TAAD) is responsible for significant morbidity and mortality, with conservative estimates ranging up to 15,000 deaths/year in the USA alone [14]. Advances in genetics and medical screening continue to increase the number of TAADs diagnosed each year, which suggests that many prior dissection or rupture related deaths may have been falsely ascribed to other causes, including heart attack. In fact, among patients suffering out-of-hospital cardiac arrest undergoing peri-mortem diagnostic CT scan, a staggering 8.07% had succumbed to undiagnosed aortic dissection [15]. Even more troublesome is that thoracic aortic dissections are the cause of death in over 5% of all young people suffering sudden cardiac death [16]; that is, these lethal conditions affect young and old alike. There is clearly a pressing need for increased understanding.

Notwithstanding concerns with aortic diameter as a reliable metric of risk for acute aortic syndromes [17–19], this easily measured quantity yet continues to play a critical role in clinical decisions [20]. Because the aorta dissects or ruptures only when the mechanical stress exceeds the strength of the wall, many authorities also continue to suggest a similar need to calculate and compare values of wall stress [21–23]. For reasons noted above, however, there has been considerable variation in the measurement of aortic root diameter and no truly reliable method to estimate wall stress. We believe that in the future, with the advancement of methods for determining material stiffness and strength properties, having finite element computations in the decision loop will potentially provide a promising aid to clinical patient evaluation. However, in the absence of material parameters and image-based finite element computations in everyday practice, the presented enhanced Laplace method addresses a pressing need for mechanistic tools that inform decisions related to the Sinuses of Valsalva.

Herein, we suggest an extended law of Laplace that provides a simple, universal estimate of wall stress in the aortic root that overcomes challenges with both the inappropriate use of the classical law of Laplace and insufficient information on patient-specific material properties needed for image-based finite element calculations. Moreover, this simple derivation revealed a reliable, easily measured, metric—the Laplace diameter—that promises to help delineate risk across different classes of aneurysms based on medical images of the aortic root. The intra-observer and inter-observer variabilities for our Laplace technique of aortic root measurement fall well within the ranges usually found for traditional measurements of aortic diameter, where “Variations in AAA measurement of 0.5 cm or more are not uncommon…” [24–26].

As illustrative results, we found that this Laplace diameter was significantly larger than three other commonly used measures of aortic root dimensions, suggesting further that the prior measurements of diameter led to underestimating wall stress. Importantly, the Laplace diameter did not correlate strongly with patient height in the three aneurysmal groups considered–non-syndromic, bicuspid, and Marfan. Rather, the Laplace diameter correlated well with the cross-sectional area at the aortic root, consistent with it being primarily a measure of size (R^2^ = 0.96, non-dilated group). As expected, the other measurements are also related to the size of the root and correlate rather well with cross-sectional area (R^2^ = 0.95 for depth-to-depth and depth-to-commissure measurements, and R^2^ = 0.93 for coronal measurements within the non-dilated group). With practice, determining the Laplace diameter becomes a simple method to quantify aortic size, providing an appropriate indicator of severity of wall stress (see accompanying video, demonstrating application of the Laplace measurement technique). These Laplace metrics, however, depend on the existence of certain symmetries within the aortic root. Of the cases considered, the aortic root in patients with bicuspid aortic valve exhibited a lack of root symmetry, thus necessitating careful evaluation of root shape, not just size, and warranting caution in the use of these metrics in these cases. In cases of symmetry, however, the extended law of Laplace and the Laplace diameter upon which it is based provides quick, straightforward assessments. It is suggested that these metrics can be added to others to increase risk stratification in regression-based predictive models [27]. Finally, we hope that the proposed method would complement previous efforts in measurements of the aortic root structure [28, 29] and contribute to more uniform analysis of the root dimensions.

## Conclusion

In this study, we addressed head-on issues with discrepant methods of measurement of the aortic root—issues originating from its non-circular, cloverleaf shaped cross-section. Using fundamental mathematical calculations, we extended the classical law of Laplace specific for the cloverleaf shaped cross-section at the aortic root. This work exploits basic force balances and particular symmetries in geometry; it was thus not an experimentally determined modification of the law of Laplace, but rather a purely mathematical derivation. Like the classical law of Laplace, this extended law is universal, thus determining radially-averaged circumferential wall stress in aortic roots having two or more planes of symmetry, independent of specific material properties. We then confirmed the validity of the mathematical derivation using computational finite element analyses with an idealized root geometry defined by an epitrochoid and other symmetric geometries such as ellipses, triangles, and diamonds and various values of material parameters for both linear and nonlinear material descriptions. The wall stresses calculated from the finite element computations and the extended law of Laplace (Eq. 1) agreed with high precision. In the cloverleaf cross-sectional geometry of the Sinuses of Valsalva, the extended law of Laplace introduces the diameter of the circumscribed circle as the primary dimension related to wall stress. The Laplace diameter was larger than the sinus-to-commissure, the mid-sinus-to-mid-sinus, and the diameter in the coronal plane, suggesting that the other measurements would underestimate the wall stress. Regardless, this Laplace diameter offers a new, theoretically motivated approach to measure and compare aortic root dimensions uniformly across centers.

## Perspectives/clinical competencies

This manuscript analyzes in detail multiple factors involved in achieving well motivated and consistent measurements of the aortic root. The root portion of the aorta is anatomically complex and is quantification has been problematic in terms of intra- and inter-institutional methodology, subject to high variability and little uniformity. The geometric analyses herein should be of use to clinicians of all levels of experience. The derivation of an extended law of Laplace in this manuscript applies basic geometric and mechanical principles and more accurately represents maximal wall stress. This extension of Laplace has implications in terms of optimal methods of measuring the “diameter” of a cloverleaf shaped cross-section. We hope that this analysis and these recommendations may encourage standardization of measurements of the aortic root in a geometrically justified fashion.

## Supplementary Information

Below is the link to the electronic supplementary material.

Supplementary Video S1. Application of the new Laplace diameter measurement technique to patient CT scans. (MP4 101211 KB)

Supplementary Methods and Results on Mathematical and Computational Modeling (DOCX 20 KB)
