# Supplementary material for: Extended law of laplace for measurement of the cloverleaf anatomy of the aortic root
Source: Int J Cardiovasc Imaging. 2023 Apr 12;39(7):1345–56. doi: 10.1007/s10554-023-02847-5 (PMC10250276; doi:10.1007/s10554-023-02847-5)
Supplement: Supplementary file 2 — Supplementary Methods and Results on Mathematical and Computational Modeling (DOCX 20 KB) [file 10554_2023_2847_MOESM2_ESM.docx]

Supplementary Information for the Article:

**Extended Law of Laplace for Measurement of the Cloverleaf Anatomy of the Aortic Root**

Ehsan Ban PhD^1^, Paris-Dimitrios Kalogerakos MD, PhD^2^, Ramak Khosravi MD, PhD^1^, Bulat A. Ziganshin MD, PhD^2^, Hesham Ellauzi MD^2^, Abhay B. Ramachandra PhD^1^, Mohammad A. Zafar MD^2^, Jay D. Humphrey PhD^1^, John A. Elefteriades MD, PhD (hon)^2^

^1^Department of Biomedical Engineering, Yale University and

^2^Aortic Institute, Yale School of Medicine, New Haven, CT, USA

Address for Correspondence:

John A. Elefteriades, MD, PhD (hon)

Aortic Institute at Yale New Haven

CB-3

789 Howard Ave.

New Haven, CT 06510

Phone: 203-785-2551

FAX: 203-785-3552

e-mail; [john.elefteriades@yale.edu](mailto:john.elefteriades@yale.edu)

ORCID ID: 0000-0001-6255-8139

**Supplementary Methods and Results**

***Brief mathematical derivation of an extended law of Laplace***

For readers having interest, we summarize the derivation of Eq. 1 from the main text here. Similar to the classical Laplace relation, the derivation relies on a hypothetical free body that includes a portion of the aortic wall between a sinus depth, $D$, and an adjacent commissure, $C$, as well as the blood pressure (*BP*) acting on this portion of the wall (Fig. 3). Two planes of symmetry are present within this portion of the wall (dashed lines in Fig. 3B), which coincide at the center $O$. The derivation includes two steps. First, finding a special point within each symmetry plane that can be used to find wall tension at any point on the wall (noting that wall tension divided by local wall thickness yields mean wall stress). Second, noting that these points coincide at the intersections of the symmetry planes, $O$, the distance from this point multiplied by blood pressure gives wall tension.

The forces acting on this free body result from blood pressure acting on the segments corresponding to the planes of symmetry and the wall tensions at the depth and commissure (dark arrows in Fig. 3B, Main Text). If we consider another adjacent segment, the reflection of the previous segment with respect to a symmetry plane and to the left of the wall segment, it will experience a reaction to the wall tension at $D$, equal and opposite to that of the segment in Fig. 3B. However, the action-reaction forces are also equal because of reflective symmetry. For the two forces to be equal and opposite, the component perpendicular to the symmetry plane must equal zero.

We can find a point $Q$ residing on the plane of symmetry passing through $D$ such that wall tension at this depth equals *BP* × distance from depth to $Q$. Since the pressure over the distance $DQ$ cancels out tension at that depth, connecting $Q$ to any other point on the wall, say $A$, will result in a distance whose product by *BP* equals wall tension at that point – this results from considering a free body defined by $D$, $Q$, and $A$, and the same arguments as the original free body considered. Hence, we find one special point. This property would exist for a commissure $C$ as well. On the other hand, by considering similar arguments about $C$, including the symmetry and action-reaction property of the forces, the force acting on the wall at $C$ is perpendicular to the plane of symmetry at $C$ as well. Therefore, a point $P$ can be found along the plane of symmetry at $C$ such that *BP* × distance from $P$ to $C$ equals wall tension at $C$. Then, *BP* × distance $PC$ cancels out tension at the commissure and tension at any point within the wall equals *BP* × distance from $P$. Because wall tension at every point over the wall is proportional to the distance from each of the two points, $Q$ and $P$, this implies equal distances. Hence, the two points must coincide – namely, the only point shared by the two planes of symmetry coincide at $O$. Therefore, the tension within the wall at any point equals *BP* × distance from $O$. The wall stress that opposes the pressure can be estimated as the tension at the wall divided by wall thickness, thus we arrive at Eq. (1) for wall stress. The equality holds at the points where the planes of symmetry coincide; at the other points, the equation gives the stress from the tensions that oppose blood pressure and approximates wall stress.

It is noted, in addition, that like the classical law of Laplace, this extended relation can be used to estimate wall stress at any time during the cardiac cycle (given the pressure at that time) under the assumption of quasi-equilibrated states given that the inertial loads tend to be negligible. Hence, the results can be used equally well for gated or non-gated CTs, noting that the investigators should specify the imaging method to facilitate comparisons across studies. Our results are for CTs gated to diastole, with the valve closed.

***Finite element model***

The so-called finite element method is a powerful computational approach for solving problems in mechanics having complex geometries and/or applied loads. We performed an extensive set of in-plane finite element numerical computations to verify the validity of the mathematical derivation of the extended law of Laplace. We used both FEniCS [1] and Abaqus [2] for computations. We performed implicit quasi-static analyses that solved the momentum balance equation. These computations provided a method for ensuring force balance in a complicated geometry such as the section of the aortic root.

Specifically, our finite element models consisted of triangular elements covering an idealized cloverleaf shape constructed by the equation of an epitrochoid. In the complex plane, with points $z=x+yi$ (with $i$the imaginary number $\sqrt{-1}$), mapping $z$ from the unit circle onto $z+0.2 z^{4}$ generated an idealized epitrochoid shape, which could then be transformed to produce cloverleaves of various sizes (Fig. 4A, Main Text). For completeness, other shapes, such as ellipses, triangles and diamonds, having 2, 3, or 4 planes of symmetry were tested as well. Half of the symmetric shapes were modeled, and symmetric boundary conditions were used. Blood pressure was prescribed as traction boundary conditions. We considered representative values of pressure, 80, 93, 120, and 150 mmHg. A few degrees of freedom where constrained to remove rigid body motions and enable solution of the equilibrium equations in a matrix form. Whereas Laplace relation(s) are universal (independent of the mechanical behavior of material), one must prescribe material properties in finite element simulations. We used both linearly elastic and nonlinearly elastic (neo-Hookean) models as test cases. The resulting stresses in the wall were evaluated by summing reaction forces over radial sections made through the vessel wall and by division of the resulting traction force by the corresponding cross-sectional area. In the tested geometries, this calculation results in a stress that opposed blood pressure, approximating the maximum principal stress experienced by the tissue. Validation was performed by picking multiple points around the perimeter of the root’s section. Finite element model results were compared with values obtained by the input of various radii into the extended Laplace relationship (Eq. 1).

Finally, note that in cases of highly asymmetric roots, not present in our data, finite element calculations suggested that the extended Laplace law provides a conservative estimate of wall tension.

***An illustrative example***

As an illustrative comparison between the classical and extended laws of Laplace, consider the following example. Use of the classical Laplace law requires a diameter for a circular cross-section as an input. Within the hypothetical root shape represented in Fig. 4A (Main Text), consider a depth-to-commissure measurement to estimate diameter for the classical Laplace law. The resulting stress values (assuming equal wall thickness) differ from finite element computations by ~16%. The extended Laplace law, by comparison, resulted in a near exact comparison; computational values differed from Eq. 1 by less than 0.01%.

**Supplementary References**

1. Alnæs M., Blechta J., Hake J., et al. The FEniCS Project Version 1.5. Archive of Numerical Software 2015;3(100). Doi: 10.11588/ans.2015.100.20553.

2. Hibbett., Karlsson., Sorensen. ABAQUS/standard: User’s Manual. vol. 1. 1998.
